# Potent long-acting rhFGF21 analog for treatment of diabetic nephropathy in db/db and DIO mice

**DOI:** 10.1186/s12896-017-0368-z

**Published:** 2017-07-04

**Authors:** Longwei Zhao, Huiyan Wang, Junjun Xie, Zilu Chen, Xiaokun Li, Jianlou Niu

**Affiliations:** 1 School of Pharmacy, Wenzhou Medical University, Wenzhou, 325035 China; 2 Laboratory Medical College, Ji Lin Medical University, Ji Lin, 132013 China

**Keywords:** PEGylated rhFGF21, Half-life, Diabetic nephropathy

## Abstract

**Background:**

Fibroblast growth factor 21 (FGF21) is an endocrine-acting hormone that has the potential to treat diabetic nephropathy. However, development of FGF21 into a therapeutic has been hindered due to its low intrinsic bio-stability. In our previous study, we have developed a recombinant human FGF21 (rhFGF21) variant by site-directed mutagenesis and solid-phase PEGylation, which retained its biological function. The aim of this study is to elucidate whether the therapeutic effect of PEGylated rhFGF21 (PEG-rhFGF21) on diabetic nephropathy in DIO (diet induced obesity) mice is more significant than rhFGF21 in vivo.

**Results:**

After administration with rhFGF21 and PEG-rhFGF21 for 2 months, biochemical data and histological examination showed that PEG-rhFGF21 significantly lowered lipid levels in the kidney, decreased urine albumin/creatinine ratio (ACR) and improved mesangial expansion, demonstrating that PEG-rhFGF21 was more efficacious in ameliorating functional and morphological abnormalities induced by diabetic nephropathy in db/db and DIO mice.

**Conclusions:**

Our findings suggest that PEG-rhFGF21 treatment is more effective in treating diabetic nephropathy than rhFGF21, through enhancements of systemic metabolic alterations and anti-inflammatory mechanisms. These findings help provide a theoretical basis to develop more long-acting and efficacious protein drugs for diabetic nephropathy.

## Background

Fibroblast growth factors (FGFs) carry out their pleiotropic functions in development, transformation, tissue homeostasis and metabolism [1–3]. As a novel member of FGFs, FGF-21 is preferentially expressed in the liver, binds to a membrane-bound co-receptor, beta-klotho, and selectively activates FGFR1c isoforms in metabolic tissues [4–6]. In 2005, Alexei et al. firstly confirmed that FGF21 as a potent metabolic regulator can lower the levels of blood glucose and triglyceride when therapeutically administered to diabetic rodents [7]. Subsequent studies demonstrated that FGF21 shows more beneficial effects, such as, suppressing hepatic glucose production, reversing hepatic steatosis, increasing expenditure, improving insulin sensitivity in diet-induced and genetically modified diabetic animal models [8–10]. In addition, FGF21 also shows a potent weight loss effect and has potential to act as an anti-obesity therapeutic [11]. Interestingly, a recently study suggested that daily injection for 3 months at a dose of 25 μg/kg/d FGF21 protects against renal injury though both improvement of systemic metabolic alterations and anti-fibrotic effects in type 2 diabetic nephropathy.

However, the outstanding pharmaceutical profiles of FGF21 are often accompanied by its poor pharmacokinetic properties, thereby reducing its therapeutic efficacy [12]. Recent clinical trials of an FGF21 variant did not significantly reduce the glucose level, probably due to its short serum half-life in vivo [13]. Polyethylene glycol modification (PEGylation) is regarded as one of the most successful strategies to improve pharmacokinetic properties of protein drugs by increasing their in-vivo circulation half-life, which implies prolonged therapeutic effects and reduced dosage [14–17]. In our previous study, we’ve developed PEG-rhFGF21 by strategically introducing cysteine residues via site-directed mutagenesis, followed by PEGylation using PEG-maleimide. Compared to the native form, PEG-rhFGF21 afforded a significantly long effect on reducing blood glucose and triglyceride levels in type 2 diabetic mice, suggesting that PEG-rhFGF21 is a more promising and effective anti-diabetic drug candidate than rhFGF21 [18].

In the present study, we have evaluated the efficacy of this engineered PEG-rhFGF21 for treatment of diabetic nephropathy in db/db and diet-induced obesity (DIO) mice. Biochemical data and histological examination revealed that PEG-rhFGF21 significantly lowered albuminuria levels and improved mesangial expansion in db/db and DIO mice, demonstrating that PEG-rhFGF21 was more efficacious in ameliorating functional and morphological abnormalities induced by chronic diabetic injury.

## Methods

### Materials

C57BL/6 J, C57BL/6KS and C57BL/6KS db/db mice were purchased from Chinese Academy of Science-Shanghai Laboratory Animal Center. PEGylated rhFGF-21 and rhFGF21 were produced by Key Laboratory of Biotechnology and Pharmaceutical Engineering of Zhejiang Province, Wenzhou Medical University. The Ni-NTA resin column and Q-Sepharose FF column, and AKTA purifier were purchased from GE Healthcare (Piscataway, NJ, USA); all other chemicals and reagents used in these experiments were of analytical grade and purchased from Sigma-Aldrich Co.

### Animal experiments

C57BL/6KS db/db mice fed with standard diet were randomly divided into four groups and they were treated by physiological saline (*n* = 6), 20 kDa PEG-Maleimides (*n* = 6), 25 μg/kg rhFGF21 (*n* = 6) and 25 μg/kg PEG-rhFGF21 (*n* = 6) respectively. After fed with high fat diet for 3 months, C57BL/6 J mice with blood glucose levels more than 180 mg/dl were considered to be efficient DIO mice. DIO mice were randomly divided into three groups and treated by physiological saline (*n* = 10), 20 kDa PEG-Maleimides (*n* = 10), 25 μg/kg rhFGF21 (*n* = 10), and 25 μg/kg PEG-rhFGF21 (*n* = 10). C57BL/6 J mice (*n* = 10) fed with a standard diet were treated with physiological saline (*n* = 10) and served as a sham control. RhFGF21 and PEG-rhFGF21 were administered every three days for 2 months by intraperitoneal injection. We selected this dose of rhFGF21 based on the data from previous study [19].

Plasma glucose levels were measured using a glucose oxidase-based method, Plasma insulin levels were measured with an enzyme-linked immunosorbent assay (ELISA) kit (Linco Research, St. Charles, MO, USA). RhFGF21 concentration was measured with an ELISA kit (R&D Systems Inc, Minneapolis, MN, USA). The homeostasis model assessment index (HOMA-IR) was calculated using the formula of fasting glucose (mM) [20]. Plasma triglyceride and cholesterol analyses were performed using a GPO-Trinder kit (Sigma-Aldrich, St.Louis, MO, USA). Urinary levels of 8-isoprostane were measured with an ELISA kit (Cayman Chemical, Ann Arbor, MI, USA). Lipids from renal cortical tissues were extracted as described by Bligh and Dyer [21]. Total cholesterol and triglyceride contents were measured using a commercially available kit (Wako Chemicals, Richmond, VA, USA). The lipid hydroperoxides (LPOs) in kidney was determined by using an LPO assay kit (Cayman Chemical). Urinary albumin concentrations were determined by competitive ELISA (Shibayagi, Shibukawa, Japan) and then corrected by urinary creatinine concentrations. After 2 months of treatment, mice were euthanized under anesthesia by intraperitoneal injection of sodium pentobarbital (50 mg/kg). Kidneys were weighed and subsequently snap frozen in liquid nitrogen. Animal care and all animal experiments conformed to the Guide for the Care and Use of Laboratory Animals provided by U.S. National Institutes of Health and approved by the Animal Care and Use Committee of Wenzhou Medical University, China. ARRIVE guidelines have been adhered to.

### Analysis of gene expression by real-time quantitative PCR

Total RNA was extracted from the renal cortical tissues with TRIzol reagent (Invitrogen, Carlsbad, CA). After quantification using a Nanodrop ND-1000 spectrophotometer, 1 μg total RNA was used to synthesize the first-strand complimentary DNA (cDNA) using reverse transcription kit (Promega, WI) following the manufacturer’s protocol. Quantitative real-time RT-PCR was performed for 2 min at 95 °C and a total of 40 cycles of 10 secs at 95 °C and 30 sec at 60 °C. Samples were finally heated to 95 °C to verify that a single PCR product had been obtained. The ratio of the expression level of each gene to that of β-actin level (relative gene expression number) was calculated by subtracting the threshold cycle number of the target gene from that of β-actin and raising two to the power of this difference.

### Histopathological evaluation and immunohistochemistry

The treatment of kidney and glomerular mesangial expansion’s score were done based on the previous report [22]. For histopathological evaluation, renal tissues were sliced into 5 μm sections and fixed overnight in 4% paraformaldehyde and embedded in paraffin. After deparaffinization and rehydration, the paraffin sections (5 μm) were subjected to periodic acid–Schiff staining (PAS). The immunohistochemically staining was performed as previous study. For evaluation of immunohistochemically staining for type IV collagen, TGFβ1, fibronectin and laminin, glomerular fields were graded semi-quantitatively using a high-power field (HPF) containing 50–60 glomeruli and an average score was calculated as described previously. A pathologist carried out the histological examinations in a blinded manner.

### Pharmacokinetic evaluation of PEG-rhFGF21 and rhFGF21 in vivo

The in vivo pharmacokinetic properties of the two forms of rhFGF-21 were analyzed by intravenous (IV) injecting a single dose of 25 μg/kg either PEG-rhFGF21 or rhFGF21 in DIO mice, followed by measuring the dynamic levels of the two forms of rhFGF21 in the plasma at the indicated time points using ABC-ELISA method as previous report [18].

### Measurement of exogenous rhFGF21 Concentration in the Kidney

To determine the retention levels of PEG-rhFGF21 and rhFGF21 in the kidney, rats were anesthetized after two months’ treatment and kidneys were homogenized in lysis buffer and centrifuged at 12,000 g at 4 °C for 15 min. Total protein concentration was determined by bicinchoninic acid (BCA) protein assay kit. RhFGF21 concentrations were measured by ABC-ELISA.

### Western blot analysis

For western blotting, 40ug of protein was electrophoresed on a 10% SDS-PAGE gel. Proteins were transferred onto a polyvinylidene difluoride membrane, and the membranes were hybridized in blocking buffer at 4 °C overnight with mouse monoclonal anti-CD68 antibody (1:1000, Cell Signaling Technology), rabbit polyclonal anti-TNF-α antibody (1:500; Santa Cruz Biotechnology, Santa Cruz, CA, USA), anti-IL-6 antibody (1:500, Santa Cruz Biotechnology, USA), β-actin (1:500, Santa Cruz Biotechnology, USA), mouse monoclonal anti-fibronection antibody (1:1000, Cell Signaling Technology), mouse monoclonal anti-Type IV collagen antibody (1:1000, Cell Signaling Technology), mouse monoclonal anti-laminin antibody (1:1000, Cell Signaling Technology). The membranes were subsequently incubated with horseradish peroxidase-conjugated secondary antibody (1:5000 dilution) for 60 min at room temperature and visualized using enhanced chemiluminescence (ECL) reagents (Bio-Rad, Hercules, CA). The relative amount of the proteins was then analyzed using Image J analysis software version 1.38e (NIH, Bethesda, MD) and normalized against their respective controls.

### Statistical analysis

A nonparametric analysis was used for all data analysis due to small sample size. Results were expressed as the mean standard error of the mean (SEM). Multiple comparisons were done using Wilcoxon rank sum tests and Bonferroni correction. A Kruskal-Wallis test compared more than two groups, followed by a Mann–Whitney *U*-test, using a microcomputer-assisted program with SPSS for Windows 10.0 (SPSS, Chicago, IL, USA). P‹0.05 was considered statistically significant.

## Results and Discussion

### The hypoglycemic effects of PEG-rhFGF21 and rhFGF21 in DIO mice

**Fig. 1:**
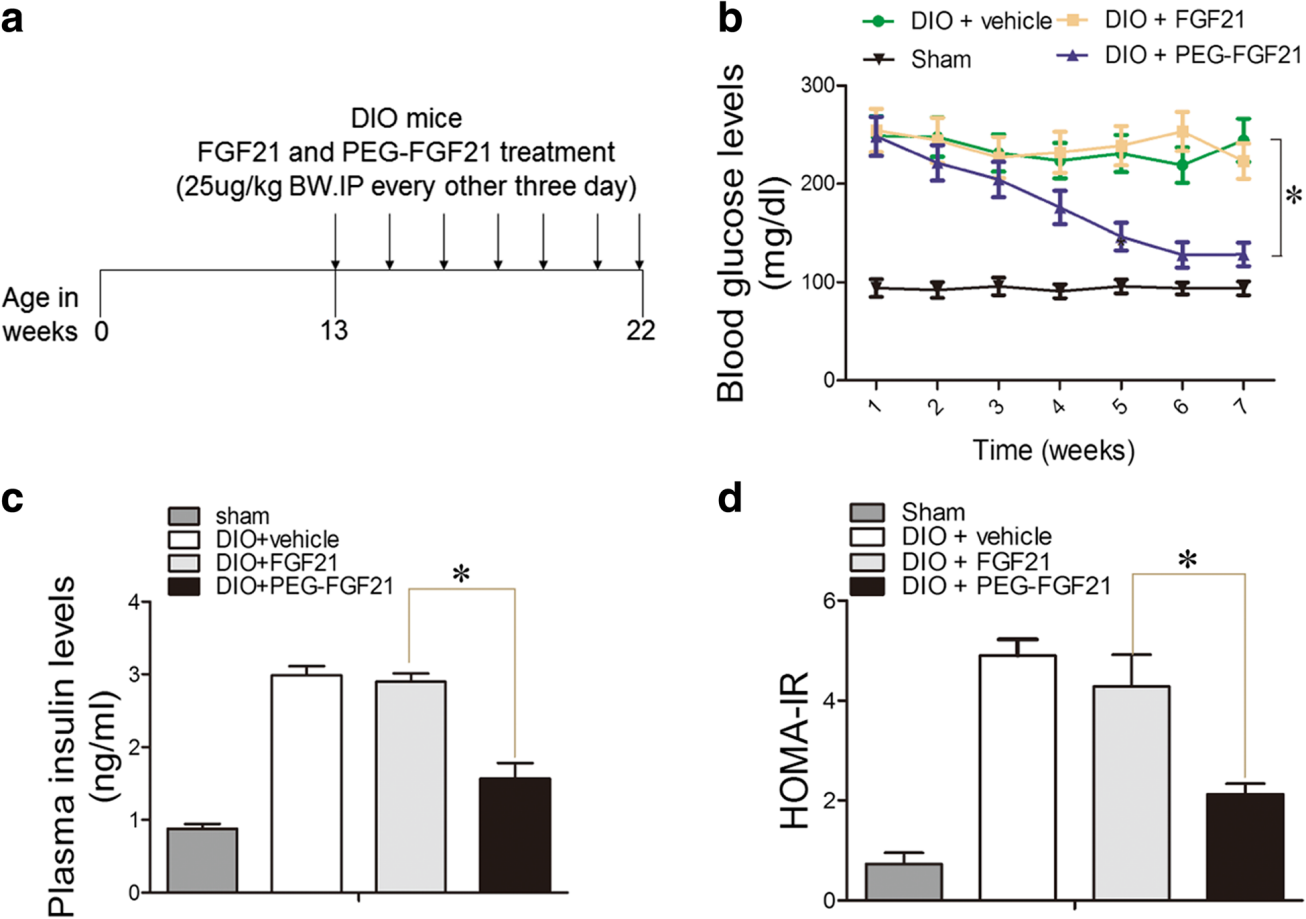
The effect of PEG-FGF21 and FGF21 on blood glucose and plasma insulin in DIO mice. FGF21 and PEG-FGF21 treatment protocol for diabetic mice wherein diabetic mice received vehicle or FGF21 and PEG-FGF21 (25 μg/kg BW, every other three days) for 10 weeks (**a**). Random-fed blood glucose (**b**) and plasma insulin levels (**c**). During chronic treatment of DIO mice with FGF21 and PEG-FGF21. Insulin resistance (IR) measured in DIO mice after 2 months of FGF21 and PEG-FGF21 treatment. IR = FINS * FBG/22.5 (**d**). Ad libitum fed mice were injected subcutaneously with 25ug/kg of FGF21, PEG-FGF21 or control vehicle (PBS) every 72 h. Values are means ± SEM. Statistics by two-tailed *t*-test: *P ≤ 0.05; vs DIO + FGF21.*n* = 10

The metabolic effects of rhFGF21 have been previously evaluated by other groups using leptin-deficient and high-fat diet-induced obesity (DIO) models [23–26]. Accumulating evidence has shown that rhFGF21 at the appropriate concentration can significantly reduce the plasma glucose levels. Previous studies have shown that a daily injection of 10–100 μg/kg rhFGF21 was required to ensure its in-vivo pharmacological activity [27, 28]. We have shown that PEG-rhFGF21 we’ve developed afforded a significantly prolonged anti-diabetic effect. To further confirm the long-acting efficacy of PEG-rhFGF21 in DIO mice, we reduce the frequency of dosing from daily to every 3 days for 2 months (Fig. 1a) and then detect the plasma glucose levels. As shown in Fig. 1b, the plasma glucose levels of rhFGF21-treated group was almost the same as that of vehicle-treated group when the frequency of administration was decreased once every three days; the plasma glucose level of PEG-rhFGF21 decreased continually within the first 6 months and returned to normal glucose level at the seventh week. We found that rhFGF21 treatment did not reduce the plasma glucose level, probably because it did not reach the minimum concentration in serum that is required to produce the desired pharmacological effect owing to its poor in vivo bio-stability and low dosing frequency. We also measured the concentration of insulin in serum, which showed that only insulin levels of PEG-rhFGF21 treated group were significantly reduced than those in the vehicle-treated group (Fig. 1c). This was consistent with the plasma glucose data, indicating that PEG-rhFGF21 administration significantly improved the insulin sensitivity in DIO mice. Homeostatic model assessment used to quantify insulin resistant and beta-cell function also confirmed that PEG-rhFGF21 significantly improved the insulin-sensitivity in comparison to rhFGF21 (Fig. 1d).

### Effect of PEG-rhFGF21 and rhFGF21 treatment on plasma lipid concentration

**Fig. 2:**
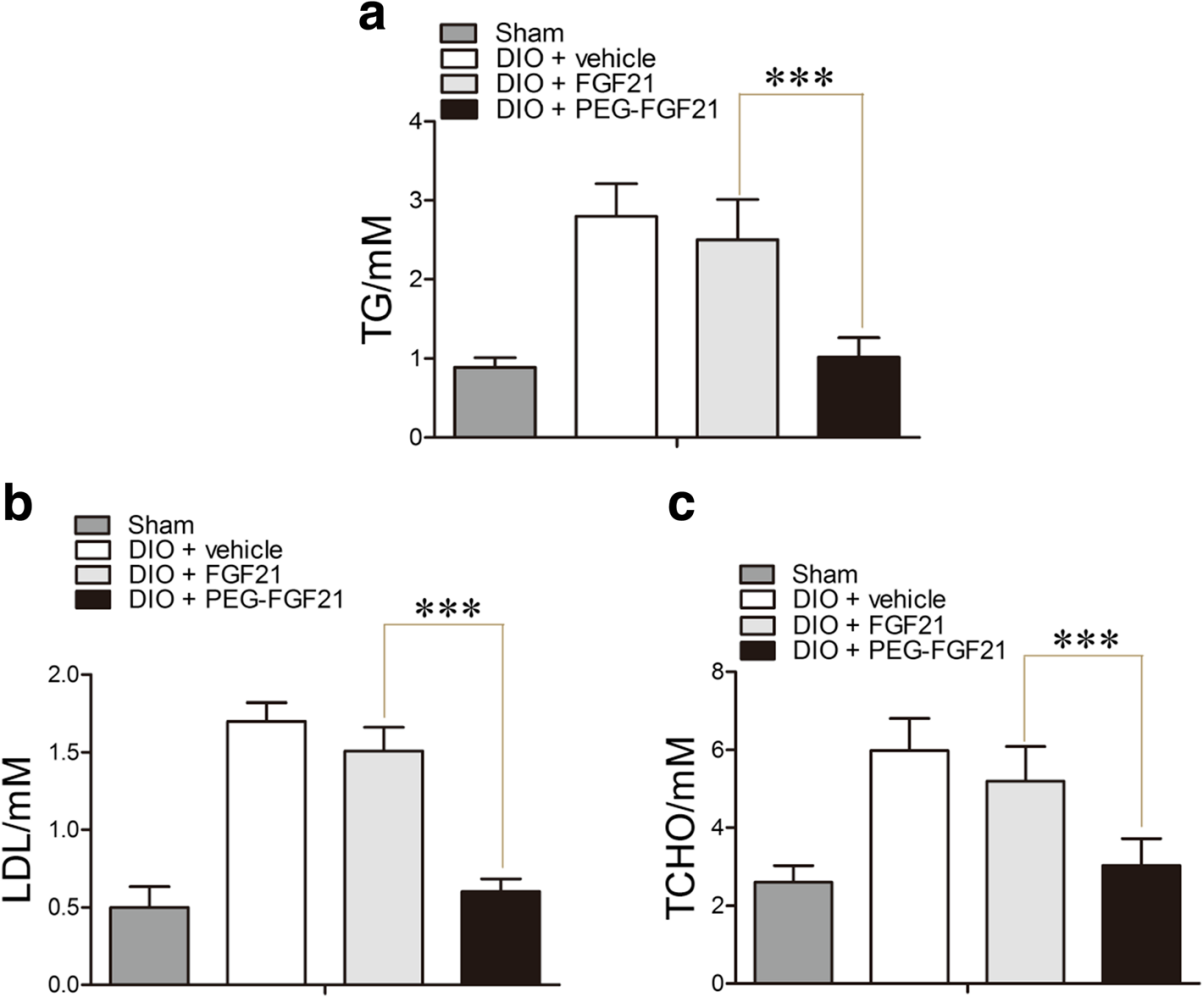
PEG-FGF21 ameliorate plasma lipid metabolism significantly compared with FGF21 in DIO mice. The level of plasma Triglyceride measured after treatment with FGF21 and PEG-FGF21(**a**), Low-density lipoprotein (LDL) content in serum (**b**), and the level of total cholesterol (TCHO) measured in serum after treatment with FGF21 and PEG-FGF21 for 2 months (**c**); Values are means ± SEM. Statistics by two-tailed *t*-test: *P ≤ 0.05; vs DIO + FGF21.*n* = 10

There have been many studies on rhFGF21 and lipid metabolism [29]. It has now been shown that rhFGF21 is capable of lowering blood lipids in animal models of diabetes [29]. In our study, we measured the levels of serum lipids,DIO diabetic mice group showed significantly increased levels of triglyceride (TG), low-density lipoprotein cholesterol (LDL) and total cholesterol (TCHO). Treatment with PEG-rhFGF21 significantly (*p* < 0.05) decreased the levels of these plasma lipids in the DIO mice, in comparison to rhFGF21 (Fig. 2).

### Effect of PEG-rhFGF21 and rhFGF21 on renal lipid profiles and urinary 8-isoprostane levels

**Fig. 3:**
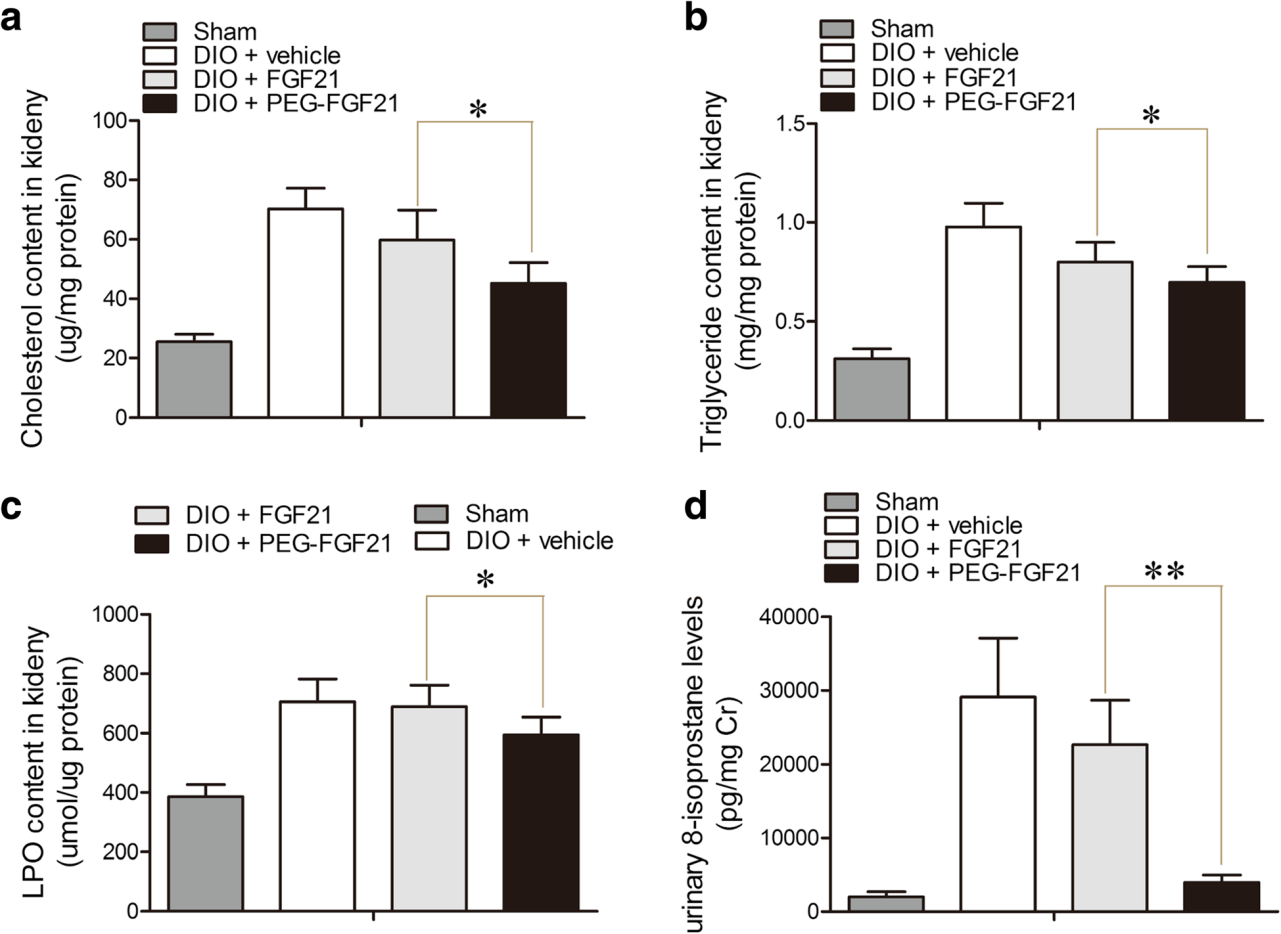
Effect of PEG-FGF21 and FGF21 on renal lipid levels in DIO mice. Cholesterol content in renal cortical tissue (**a**); Triglyceride content in renal cortical tissue (**b**); Lipid hydroperoxides (LPO) content in renal cortical tissue (**c**); 24-h urinary levels of 8-isoprostane; urinary excretion of 8-isoprostane was corrected by urinary creatinine (**d**). Values are means ± SEM. Statistics by two-tailed *t*-test: *P ≤ 0.05; **P ≤ 0.01; vs DIO + FGF21.*n* = 10

There is growing evidence to suggest that abnormal lipid metabolism and renal accumulation of lipids contribute to the pathogenesis of diabetic nephropathy [30],this triggered us to evaluate the effects of rhFGF21 and PEG-rhFGF21 treatment on the renal lipids profiles. Compared to sham mice, there was a significant elevation in the levels of renal lipids including triglyceride, LDL-cholesterol and lipid oxidation products (LOPs) in DIO mice. Treatment with rhFGF21 did not significantly reduce renal lipid levels. As expected, a remarkable decrease in the renal lipid levels was only observed in mice treated with PEG-rhFGF21 (Fig. 3a, b and c). However, this is not consistent with the previous report which showed that rhFGF21 can reduce kidney renal lipid levels in the db/db mice. This is because we reduced the administration frequency of rhFGF21 to once every three days, rather than daily injection, which probably lead to low rhFGF21 concentration in the plasma due to its short half-life. However, PEG-rhFGF21 with enhanced in vivo stability retained its hypolipidemic effect at a lower dosage.

Since PEG-rhFGF21 can reduce lipid toxicity in the kidney by alleviating kidney fat accumulation, we hypothesize that it could also improve lipid peroxidation. In order to test this hypothesis, we measured the levels of 8-isoprostane, an accurate marker of lipid peroxidation and the most extensively studied markers of diabetes-related free radical attacks [31]. As expected, urinary levels of 8-isoprostane of DIO mice were 15-fold higher than that of normal mice, and this substantial increase was abrogated by PEG-rhFGF21 treatment rather than rhFGF21 treatment (Fig. 3d). This result was in agreement with the previous study where the urinary 8-isoprostane level was markedly attenuated by rhFGF21 treatment in db/db mice.

### PEG-rhFGF21 significantly reduced serum creatinine level and urinay albumin/creatinine ratio in DIO mice

An earlier study has shown that rhFGF21 treatment significantly ameliorated functional and morphological glomerular abnormalities induced by chronic diabetic injury in db/db mice [32]. However,the clinical use of rhFGF21 for diabetic kidney injuries has been hampered by its poor in-vivo stability, a daily IP injection of 25 μg/kg rhFGF21 for 3 months was required to ameliorate renal injury in db/db mice. We have recently developed recombinant rhFGF21 variants by combination of site-directed mutagenesis and solid-phase PEGylation that retained their biological function and showed better in vivo stability. in vivo studies have shown that the anti-diabetic effects of PEG-rhFGF21 in ob/ob mice were significantly better than the non-PEGylated form. These results lead us to propose that PEG-rhFGF21 could exhibit superior renal protection over rhFGF21. Toward this, we conducted our studies using a high-fat diet induced obesity (DIO) model, because metabolic characteristics of DIO more closely resemble those of human type 2 diabetes. To test the long-acting property, we reduced the frequency of administration of PEG-rhFGF21 and rhFGF21 from daily to every three days.

**Fig. 4:**
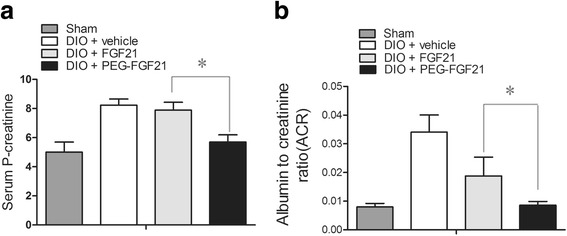
Effect of PEG-FGF21 and FGF21 on serum P-creatinine and urinary albumin/creatinine ratio in DIO mice. The levels of serum P-creatinine were measured after chronic treatment with PEG-FGF21 and FGF21 (**a**); A 24-h urine sample was collected after FGF21 and PEG-FGF21 administration and urinary albumin/creatinine ratio was determined (**b**). Statistical analysis was performed between groups at the same time periods; Data are means ± SEM; *P ≤ 0.05, vs. DIO + FGF21. *n* = 10

To determine whether PEG-rhFGF21 could retain its renal protection at low administration frequency, we examined the effect of both PEG-rhFGF21 and rhFGF21 on two important markers of renal functions (serum creatinine and urinary albumin/creatinine ratio) in DIO mice. As shown in Fig. 4a and b, compared with vehicle treated DIO mice, PEG-rhFGF21 group showed significantly reduced serum creatinine levels and urinary albumin/creatinine ratio than rhFGF21 group.

### Effects of PEG-rhFGF21 and rhFGF21 treatment on changes in histological and relative inflammatory factors levels in renal tissues of DIO and db/db mice

**Fig. 5:**
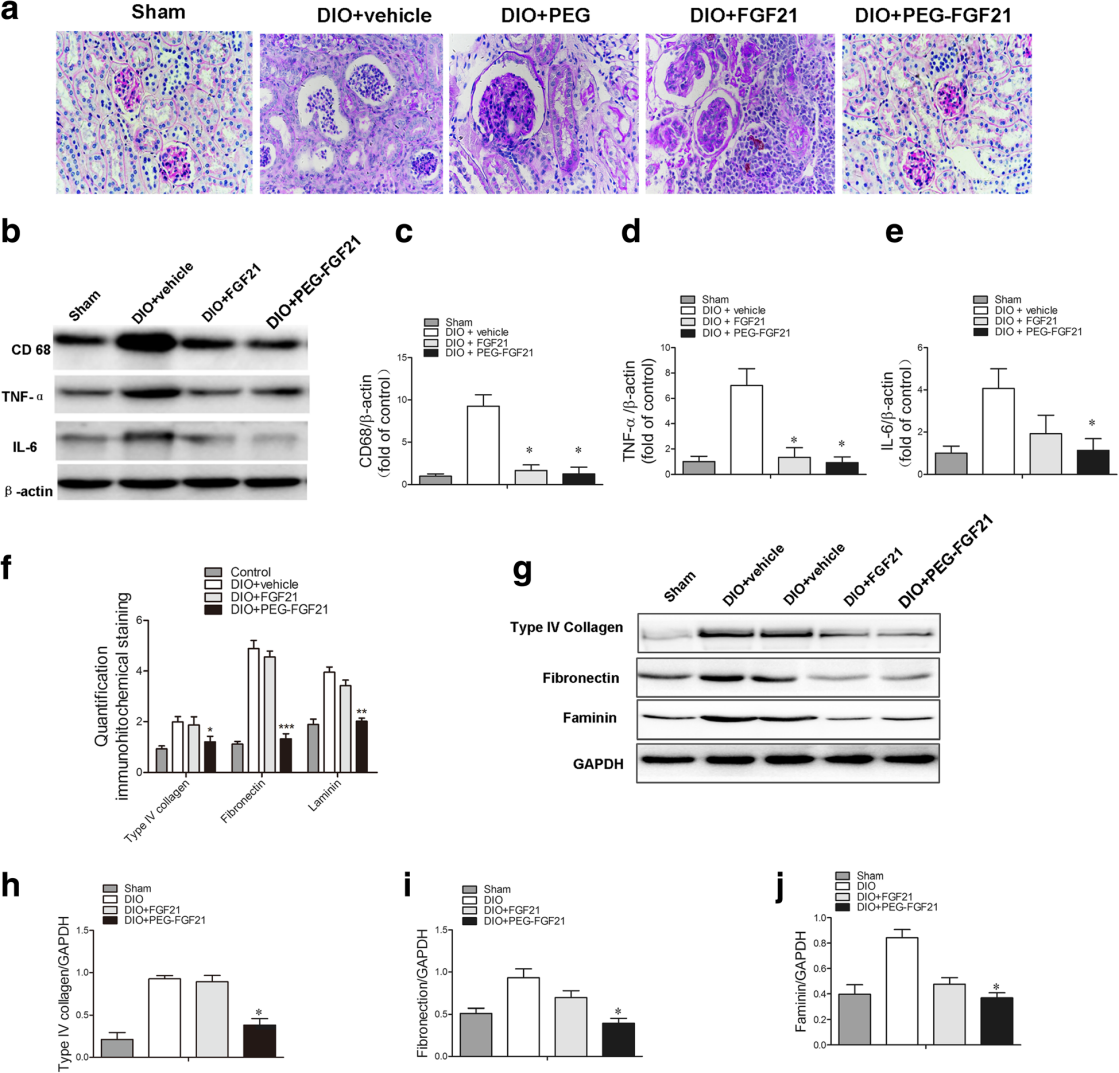
PEG-FGF21 and FGF21 administration treats established nephropathy in DIO mice of type 2 diabetes. Representative PAS staining of renal tissues of DIO mice after treatment (**a**); Western blot analysis of the levels of inflammatory factors (CD68, IL-6 and TNF-a) in renal tissues of DIO mice after treatment with FGF21 and PEG-FGF21 (**b**); Semi-quantitative analysis of CD68 (**c**), and IL-6(**d**), TNF-ɑ (**e**) expression in renal tissues after treatment with FGF21 and PEG-FGF21 by western blot; Semi-quantitative evaluation of renal collagen IV, laminin and firbonectin levels based on immnuohistochemical stains (**f**); Western blot analysis of the levels of renal collagen IV, laminin and firbonectin (**g**); Semi-quantitative analysis of collagen IV (**h**), and firbonectin (**i**), laminin (**j**) expression in renal tissues after treatment with FGF21 and PEG-FGF21 by western blot. *P ≤ 0.05 versus DIO + FGF21 treated DIO mice. *n* = 10 in each group

**Fig. 6:**
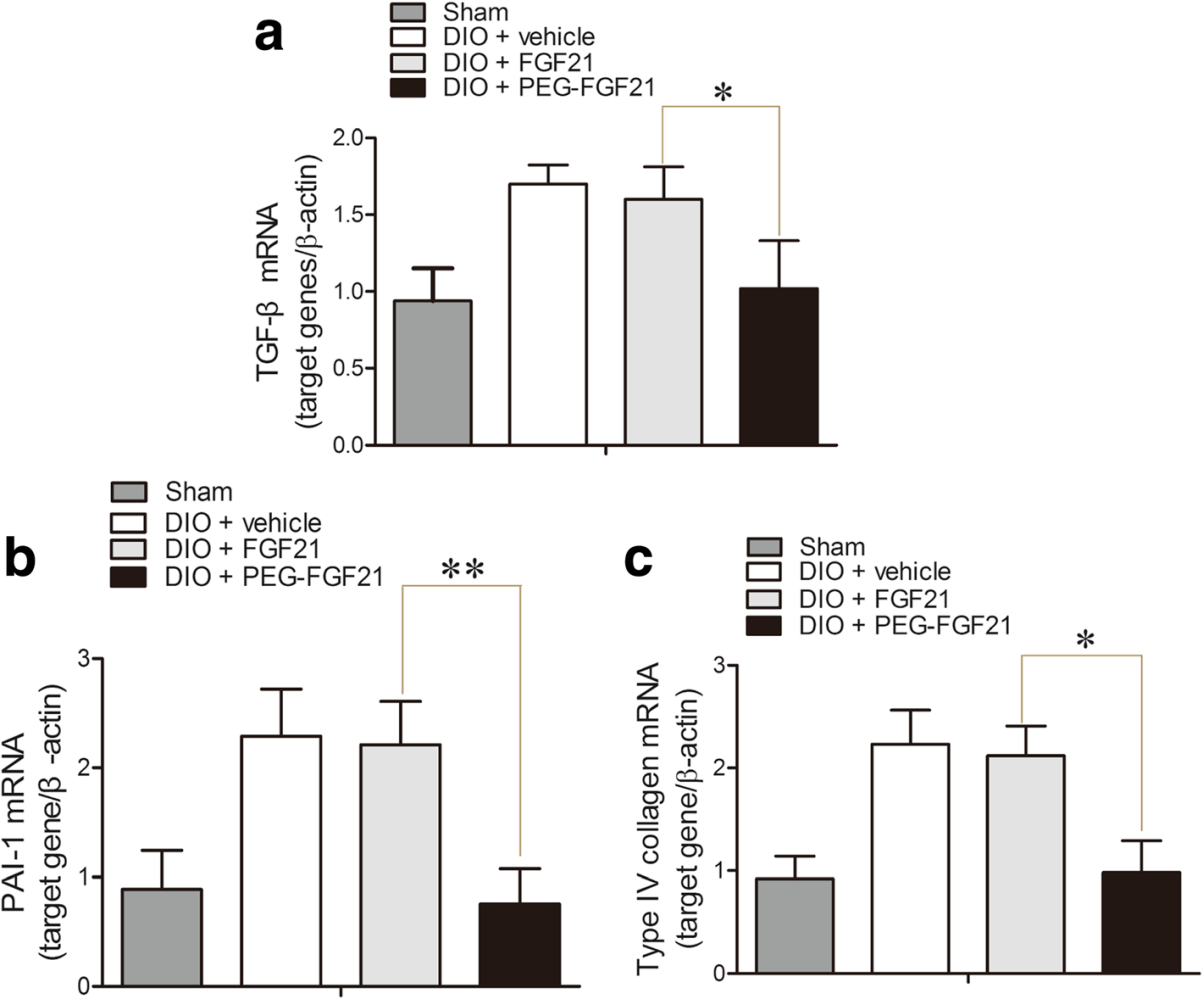
PEG-rhFGF21 ameliorates diabetes-induced renal inflammation and fibrosis. qRT-PCR analysis of the expressions of TGF-β, PAI-1 and Type IV collagen. PEG-FGF21 can significantly suppress the TGF-β, PAI-1 and Type IV collagen mRNA levels in the kidney compared with FGF21 treatment (**a**-**c**). The mRNA levels were normalized by the β-actin mRNA level. Data are means ± SEM. *P ≤ 0.05, versus DIO + FGF21 treated DIO mice. *n* = 10 in each group

Figure 5a shows the representative renal pathology in experimental animals. Consistent with marked attenuation of serum creatinine and urinary albumin/creatinine ratio, mesangial expansion and glomerulosclerotic changes were markedly improved in the PEG-rhFGF21 treatment group rather than rhFGF21 treated group by comparing with vehicle treated DIO mice (Fig. 5a). Among the many potential pathogenetic mechanisms responsible for the development of diabetic kidney disease, an inflammation mechanism has been suggested to have a major role in the development of diabetic nephropathy [33]. We observed that, consistent with these histological changes, the expression of inflammatory cytokines (such as CD68, TNF-ᾳ, IL-6) was increased in vehicle-treated DIO mice compared with the normal group, and this increase in expression of inflammatory cytokines was significantly reduced by PEG-rhFGF21 treatment (Fig. 5b-e). This observation is consistent with previous studies which have shown that rhFGF21 treatment can significantly ameliorate obesity-induced increase in proinflammatory adipocytokines such as PAI-1 and TNF-ᾳ in adipose tissue as well as enhancing anti-inflammatory [34]. Immnuohistochemical stains and western blot analysis for profibrotic markers such as type IV collagen, fibronectin and laminin showed similar tendencies with the histological and the inflammatory cytokines levels (Fig. 5f-j). Consistent with these histological changes, mRNA expression levels of type IV collagen, TGF-beta, and PAI-1 increased in vehicle-treated DIO mice compared with normal mice, and this increase in expression of profibrotic cytokine genes was significantly decreased by PEG-rhFGF21 treatment rather than rhFGF21 (Fig. 6a-c).

**Fig. 7:**
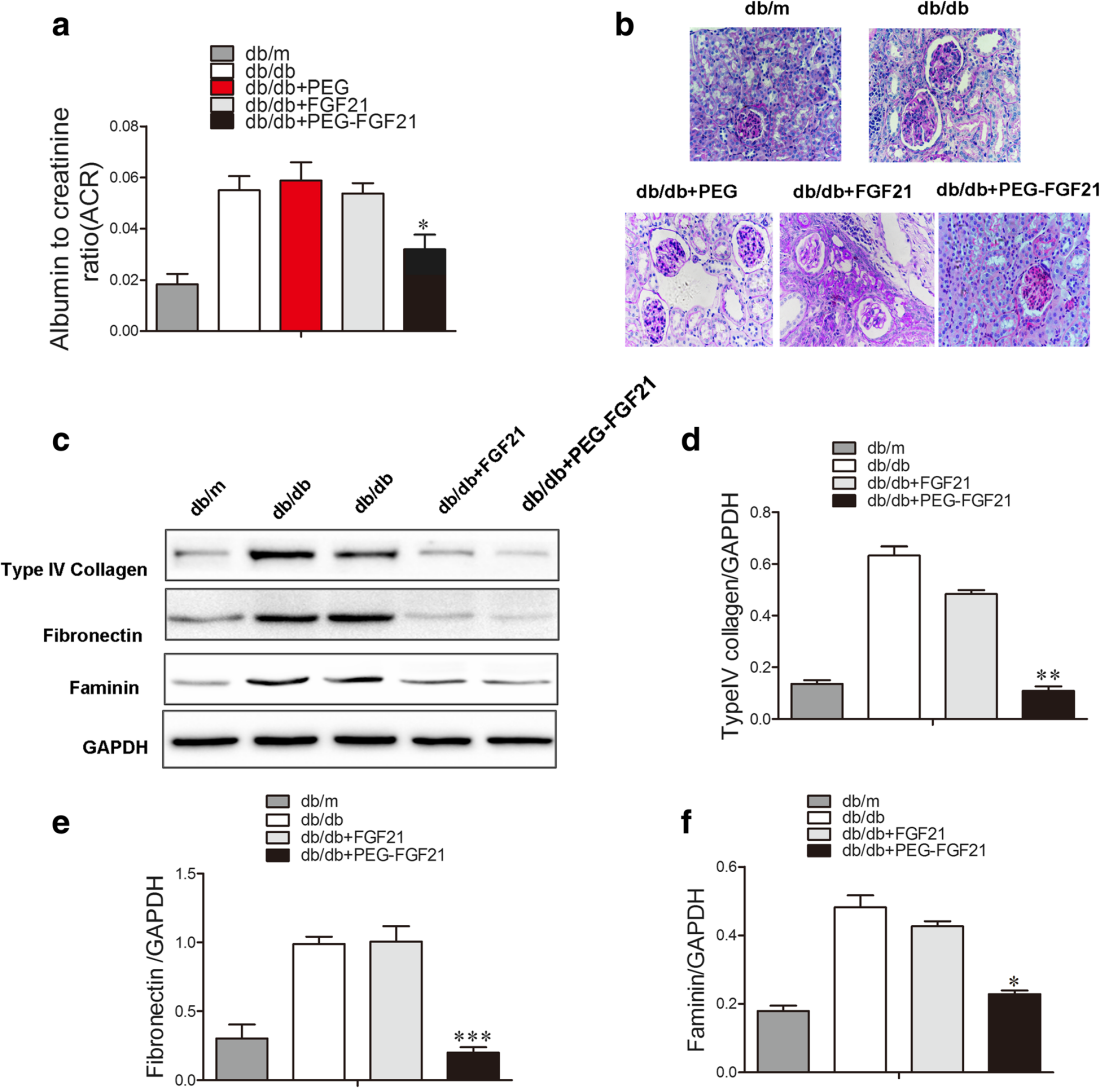
The evaluation of the renal protective effects of PEG-FGF21 and FGF21 in db/db mice. The urinary albumin/creatinine ratios (ACR) of db/db mice were determined after treatment by PEG-FGF21 and FGF21 (**a**); Representative PAS staining of renal tissues of db/db mice after treatment (**b**); Western blot analysis of the levels of renal collagen IV, fibronectin and laminin (**c**); Semi-quantitative analysis of renal collagen IV (**d**), firbonectin (**e**) and laminin (**f**) expressions by western blot after treatment with FGF21 and PEG-FGF21

We also evaluated the renal protective effects of PEG-FGF21 and FGF21 in db/db mice, another important mouse model of diabetic nephropathy. Similar with the data in DIO mice, PEG-rhFGF21 showed significantly reduced urinary albumin/creatinine ratio than rhFGF21 (Fig. 7a) and attenuated mesangial expansion and glomerulosclerotic changes (Fig. 7b) rather than rhFGF21, which confirmed the efficacy of PEG-rhFGF21 was much better that rhFGF21. In addition, PEG-rhFGF21 reduced the profibrotic makers such as type IV collagen, fibronectin and laminin (Fig. 7c-f). These results demonstrated that PEG-rhFGF21 treatment significantly ameliorated functional and morphological glomerular abnormalities induced by chronic diabetic injury in DIO and db/db mice.

### Pharmacokinetic evaluation of PEG-rhFGF21 and rhFGF21 in vivo and measurement of exogenous rhFGF21 concentration in the kidney

**Fig. 8:**
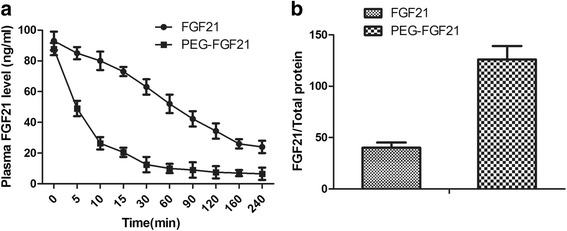
The biological stability and pharmacokinetics study of PEGylated FGF21 in DIO mice. Pharmacokinetic profiles of FGF21 and PEG-FGF21. DIO mice were injected intravenously with 25 μg/kg FGF21 and PEG-FGF21 and then blood samples were collected at the indicated time points. The concentration of FGF21 was measured using the human FGF21 immunoassay ELISA Kit(**a**); The concentrations of FGF21 and PEG-FGF21 in kidney were compared by FGF21/Total protein after treatment for 2 months (**b**)

To further confirm the reason for the superior pharmacological properties of PEG-rhFGF21 over rhFGF21, we determined the pharmacokinetic behavior of PEG-FGF21 and FGF21 and the concentrations of PEG-rhFGF21 and rhFGF21 in the kidney. Results obtained underscored where the degradation rate of PEG-FGF21 in serum was much lower than that of FGF21 in DIO mice (Fig. 8a); and the retention concentration of PEG-rhFGF21 in kidney was much higher than that of rhFGF21(Fig. 8b). Thus, it’s obvious that the excellent pharmacokinetic properties of PEG-FGF21 contribute to its better renal protection abilities.

## Conclusion

In conclusion, we found that PEG-rhFGF21 treatment can provide more stably and effectively therapeutic effects on diabetic nephropathy over rhFGF21, due to its superior pharmacokinetic properties. This is achieved through both improvement of systemic metabolic alterations and anti-inflammatory mechanisms. These findings suggest that PEG-rhFGF21 treatment may be more effective in treating renal injury than rhFGF21, which may provide a theoretical basis to develop more long-acting and efficacious protein drugs for diabetic nephropathy.

## Abbreviations

ACR: Urine albumin/creatinine ratio
BCA: Bicinchoninic acid
cDNA: Complimentary DNA
DIO: Diet induced obesity
DN: Diabetic nephropathy
ECL: Enhanced chemiluminescence
FGF21: Fibroblast growth factor 21
HOMA-IR: Homeostasis model assessment index
HPF: High-power field
IP: Intraperitoneal injection
IV: Intravenous
LDL: Low-density lipoprotein cholesterol
LOPs: Lipid oxidation products
LPOs: Lipid hydroperoxides
PAS: Periodic acid–schiff staining
PEG: Polyethylene glycol
PEG-rhFGF21: PEGylated rhFGF21
PEGylation: Polyethylene glycol modification
SEM: Standard error of the mean
TCHO: Total cholesterol
TG: Triglyceride
